# Visualisation of H_2_O_2_ penetration through skin indicates importance to develop pathway-specific epidermal sensing

**DOI:** 10.1007/s00604-020-04633-9

**Published:** 2020-11-13

**Authors:** Skaidre Jankovskaja, Anaïs Labrousse, Léa Prévaud, Bo Holmqvist, Anders Brinte, Johan Engblom, Melinda Rezeli, György Marko-Varga, Tautgirdas Ruzgas

**Affiliations:** 1 Department of Biomedical Science, Faculty of Health and Society, Malmö University, 205 06 Malmö, Sweden; 2 Biofilms - Research Center for Biointerfaces, Malmö University, 205 06 Malmö, Sweden; 3 Department of Biological Engineering, Clermont Auvergne University, 63100 Aubiere, France; 4 Faculty of Sciences, University of Montpellier, 34085 Montpellier, France; 5 ImaGene-iT, Medicon Village, 223 81 Lund, Sweden; 6 Clinical Protein Science & Imaging, Biomedical Centre, Department of Biomedical Engineering, Lund University, BMC D13, 221 84 Lund, Sweden

**Keywords:** Epidermal sensing, Hydrogen peroxide, Prussian blue, Hair follicles, Skin penetration

## Abstract

Elevated amounts of reactive oxygen species (ROS) including hydrogen peroxide (H_2_O_2_) are observed in the epidermis in different skin disorders. Thus, epidermal sensing of H_2_O_2_ should be useful to monitor the progression of skin pathologies. We have evaluated epidermal sensing of H_2_O_2_ in vitro, by visualising H_2_O_2_ permeation through the skin. Skin membranes were mounted in Franz cells, and a suspension of Prussian white microparticles was deposited on the stratum corneum face of the skin. Upon H_2_O_2_ permeation, Prussian white was oxidised to Prussian blue, resulting in a pattern of blue dots. Comparison of skin surface images with the dot patterns revealed that about 74% of the blue dots were associated with hair shafts. The degree of the Prussian white to Prussian blue conversion strongly correlated with the reciprocal resistance of the skin membranes. Together, the results demonstrate that hair follicles are the major pathways of H_2_O_2_ transdermal penetration. The study recommends that the development of H_2_O_2_ monitoring on skin should aim for pathway-specific epidermal sensing, allowing micrometre resolution to detect and quantify this ROS biomarker at hair follicles.

Graphical abstract

**Supplementary Information:**

The online version contains supplementary material available at 10.1007/s00604-020-04633-9.

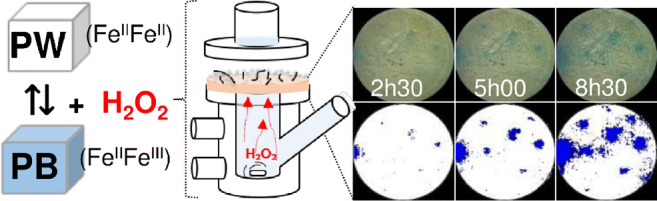

## Introduction

Robust, non-invasive, or minimally invasive sensing on skin, often referred to as epidermal sensing, is one of the tools required to realise personalised healthcare, at point-of-care units or home self-monitoring. Epidermal sensing is carried out using epidermal electronics [1–3], microneedle patches [4], attachable, stretchable, biodegradable sensors [5], etc. These developments have strongly advanced sensing of molecular biomarkers by collecting and analysing sweat right on the skin [6]. To make this approach rapid and sensitive, sweating is usually stimulated, which might impose some burden on skin [7, 8].

Epidermal non-invasive sensing that does not relay on sweating or other types of skin interrogation is less developed [9]. In such cases, the most distal skin layer, the stratum corneum (SC), is considered a tough diffusional barrier [10–14]. The biomarkers are produced in viable epidermis and dermis layers, or become available due to blood microcirculation and partition in the interstitial fluid. In all these cases, the SC severely restricts biomarker leakage out on to the surface of the skin [15]. The full complexity of structural and functional features of the skin is still poorly accounted for in epidermal sensing [16], and e.g. sensing which does not rely on sweating or other types of skin interrogation is yet to benefit from the knowledge that different molecular biomarkers have preferred transdermal permeability pathways. Targeting these pathways should improve epidermal sensing, e.g. in terms of lowered detection limits, faster detection, and better localisation of disordered skin areas.

Current technologies employed to make epidermal electronics and sensors allow manufacturing of nano- and micrometre-sized sensors, which could target specific transdermal biomarker permeability pathways, e.g. the hair follicular pathway [17, 18]. One of the restricting factors in developing pathway-specific epidermal sensing is that the permeability pathways are hard to visualise; hence, it will take some effort to convince the sensor and biosensor community that relying on these pathways is highly important for the development of superior epidermal sensing.

In this work, we show that transport of low molecular weight biomarkers, specifically H_2_O_2_, across the SC has a specific dominant pathway. Since H_2_O_2_ is a biomarker of many inflammatory disorders, this discovery might be important for development of epidermal sensing of several common skin disorders. Specifically, increased epidermal H_2_O_2_ levels are reported for vitiligo [19], polymorphic light eruption [20], skin epithelioma [21], and xeroderma pigmentosum [22]. These skin disorders are associated with skin catalase deficiency, resulting in elevated topical H_2_O_2_ concentrations. For example, it has been reported that H_2_O_2_ can reach 1 mM in the epidermis of patients affected by vitiligo [19]. Hence, robust epidermal sensing of H_2_O_2_ could provide quantitative feedback in the management of anti-inflammatory measures, e.g. by justifying choice and dosage of medications, or selecting special anti-inflammatory diets. Anti-inflammatory diet components are often specific to a particular individual [23, 24], and to monitor effects of personalised anti-inflammatory diets, an efficient and reliable personalised “inflammation sensor” is needed. We hypothesise that robust, non-invasive epidermal monitoring of H_2_O_2_ could be one of the key components of an epidermal “inflammation sensor”, allowing a quantitative assessment of the efficacy of various anti-inflammatory measures that reduce reactive oxygen species (ROS) in the epidermis. In this work, we demonstrate that H_2_O_2_ permeates skin, or more strictly SC, predominantly through a specific hair follicle pathway. We also show that Prussian white (PW) microparticles, that are relatively stable towards O_2_, might serve as a possible sensing element for routine detection of H_2_O_2_ present on the skin surface. The sensing approach and the results of this work will encourage the development of pathway-specific epidermal sensing in general and, particularly, epidermal H_2_O_2_ sensing with micrometre resolution targeting the hair follicle pathway. To the best of our knowledge, this is the first time a predominant H_2_O_2_ transdermal pathway in skin is clearly demonstrated. This work exploits an in vitro Franz cell set-up that demonstrates that initial developments of epidermal sensing can be achieved without using animal models. The results obtained in this work are also relevant to improve methods of topical application of H_2_O_2_ containing formulations for tissue oxygenation, which is one of the means to stimulate angiogenesis and healing of chronic wounds [25, 26].

## Experimental

### Materials

Sodium azide, potassium ferrocyanide (K_4_[Fe(CN)_6_]), 30% HCl, FeCl_3_, soluble Prussian blue nanoparticles, ascorbic acid, and chloroform were obtained from Sigma-Aldrich (St. Louis, MO, USA). KCl, NaCl, Na_2_HPO_4_, and KH_2_PO_4_ were purchased from Merck (Darmstadt, Germany). H_2_O_2_ 35% w/w was obtained from Alfa Aesar (Kandel, Germany), and ferricyanide (K_3_Fe(CN)_6_) from AppliChem, PanReac (Darmstadt, Germany). Phosphate buffer saline (PBS) was prepared using high-purity, 18.2 MΩ cm resistivity, Milli-Q water. PBS (pH 7.4) comprised 130.9 mM NaCl, 5.1 mM Na_2_HPO_4_, and 1.5 mM KH_2_PO_4_.

### Preparation of skin membranes

Fresh porcine ears were acquired from a local abattoir and stored at − 80 °C. The ears are residuals from food preparation, and hence ethical permission is not required. To prepare skin membranes, defrosted pig ears were cleaned with cold water and cut into stripes with a scalpel. The stripes were shaved and 0.5-mm-thick skin membranes were obtained using a dermatome (TCM 3000 BL, Nouvag, Konstanz, Germany), and circular membranes (1.6 cm diameter) were punched out from the stripes. The video of skin membrane preparation is submitted as electronic supporting information. For preparing full-thickness skin membranes, a scalpel was used to remove all tissue attached to the ear cartilage (resulting in 3–4-mm-thick skin membranes).

### Extraction of skin lipids: preparation of lipid-extracted skin membranes

In order to understand the role of the SC lipids in the H_2_O_2_ permeation mechanism, lipid-extracted skin membranes were prepared and used for H_2_O_2_ permeation studies, similarly to studies of intact skin. Lipid extraction from skin membranes was performed following a protocol published elsewhere [27]. Briefly, skin membranes were placed in 15 mL chloroform: methanol mixtures of the following compositions 2:1, 1:1, and 1:2 (v/v), and kept in each solution for 2 h. After that, the same procedure was repeated but the skin membranes were kept in each mixture for 30 min. Finally, skin membranes were left in methanol overnight, then rinsed with PBS and stored in a refrigerator (4 °C) for a maximum of 3 days until use.

### Monitoring of catalase activity in skin by using a skin-covered oxygen electrode

In skin membranes, catalase decomposes H_2_O_2_ to H_2_O and O_2_; and thus, the enzyme needs to be inhibited to allow PW/PB-based epidermal monitoring of H_2_O_2_ penetration through skin. Hence, the catalase activity and its inhibition in skin were monitored using an oxygen electrode [28]. Briefly, a skin membrane was firmly attached to the tip of the oxygen electrode by using a rubber O-ring and the assembly was inserted in an electrochemical cell filled with PBS, pH 7.4. After the baseline current of the electrode was stabilised, a defined amount of H_2_O_2_ was pipetted into the electrochemical cell (resulting in 4 mM H_2_O_2_ concentration) and the current response of the electrode was recorded (Fig. S1). When a steady-state current response was reached, the catalase inhibitor NaN_3_ (14 mM) was added to the cell. The inhibition of catalase in skin returned the electrode current to the baseline current level. An additional portion of H_2_O_2_ (resulting in 8 mM) was added to ascertain that no further decomposition of H_2_O_2_ in skin membrane occurred. The latter result ascertained that complete inhibition of catalase in skin is achieved by 14 mM NaN_3_. The solution in the electrochemical cell was continuously mixed.

### Measurement of the resistance of skin membranes mounted in Franz cells

To evaluate the skin membrane integrity and to appreciate H_2_O_2_ permeation pathways through skin, the electrical impedance of each skin membrane in the Franz cells was measured and the results were used to estimate the membrane resistance. Electrical impedance spectroscopy measurements were conducted with a skin membrane mounted in a Franz cell (PermeGear Inc.; opening diameter 0.9 cm, lower chamber volume 6 mL) equipped with four electrodes; see Fig. S2 [29]. The impedance spectroscopy measurements in the 1-Hz to 1-MHz frequency range, an applied DC voltage of 0 V, and an AC voltage amplitude of 10 mV were carried out by using a potentiostat from Ivium Technologies (Eindhoven, the Netherlands). The skin resistance was determined by fitting the impedance spectroscopy data to an equivalent circuit composed of solution resistance connected in series with skin membrane impedance (Fig. S2). Skin membrane impedance comprised parallel connection of skin membrane resistance (R_mem_) and constant phase element (CPE) (equivalent of capacitor). The fitting was done using Ivium software.

### Synthesis and characterisation of Prussian white particles

Air-stable (resistant to oxidation by O_2_ in air) PW particles were synthesised hydrothermally following a previously reported protocol [30]. Briefly, 100 mL of a 30 mM K_4_[Fe(CN)_6_] solution in water was degassed by mixing and applying vacuum for 20 min. The solution was then transferred to a Teflon-lined stainless steel autoclave (Toption Group Co., Limited, Hecheng, China) and was additionally purged with nitrogen to remove dissolved oxygen. The autoclave was sealed and maintained at 160 °C for 48 h; when room temperature was reached, the supernatant was discarded and the white particulate precipitate was collected. The particles were washed several times with 0.1 M KCl in distilled water and acetone, and finally dried under vacuum for 12 h. Commercially available PB nanoparticles were reduced to PW by washing them with saturated ascorbic acid solution in 0.1 M KCl.

The morphology of the PW particles was assessed by a scanning electron microscope (SEM; EVO LS10, Zeiss, Germany), equipped with a LaB6 filament. Briefly, different dilutions of the particles were drop-casted on Leit carbon tapes (Agar Scientific) and left to dry. Prior to SEM measurements, the samples were covered with gold, layer thickness < 10 nm, using an Auto Agar Sputter Coater (Agar Scientific, Cambridge, UK): parameters I = 30 mA, t = 40 s, and *P* = 0.08 mbar. Images were recorded in high vacuum mode, using a secondary electron detector, 15 kV accelerating voltage, and 50 pA probe current.

**Fig. 1:**
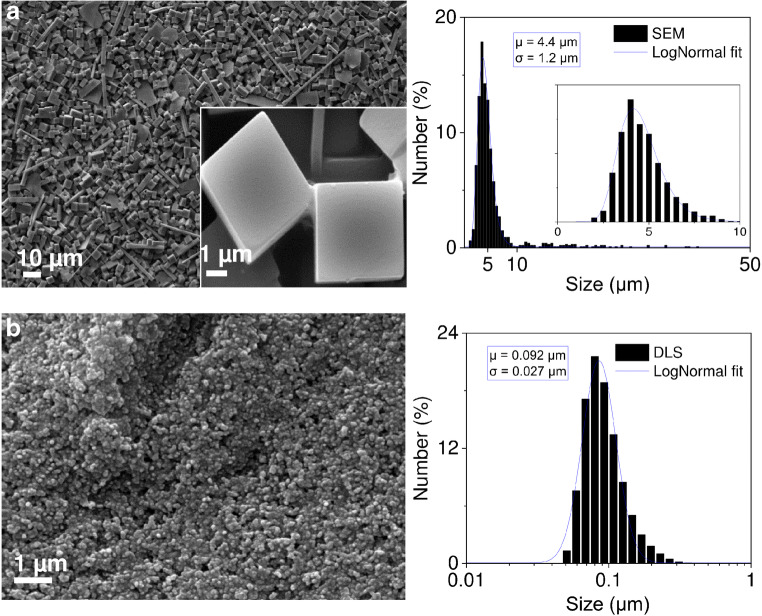
Scanning electron micrographs and particle size histograms of PW particles used to develop epidermal sensing of H_2_O_2_. Curves are fitted to LogNormal size distributions. **a** O_2_-stable PW microparticles, mainly composed of cube-like particles. **b** Commercially available PB nanoparticles, which have been reduced to PW by washing them with ascorbic acid. These PW nanoparticles exhibited high sensitivity to O_2_ (i.e. they became blue in few minutes when exposed to air). μ and σ stand for arithmetic mean particle size and standard deviation

SEM images of PW particles (Fig. 1a) were analysed with the ImageJ software to obtain the size distribution of the particles. Particle size was defined as the longest dimension measured along the PW particle. The average size of the particles was estimated after measuring the size of 990 individual particles and fitting the data to the LogNormal distribution model. Details regarding evaluation of PW particle size and shape can be found in the supporting information (Fig. S3 and Table S1).

The size of commercially available PB particles was measured by dynamic light scattering (DLS, Malvern Zetasizer Ultra, Malvern, Worcestershire, UK). The samples were prepared in deionised water. The average particle size equalled 164.7 ± 4.5 nm (*n* = 4, measurements using different dilutions and intensity weighting). To be able to compare commercially available PB particles with the synthesised PW particles, the PB size distribution determined by DLS was number-weighted and expressed in terms of number percent. The size distribution of PB particles was fitted to the LogNormal distribution model.

### Epidermal monitoring of H_2_O_2_ penetration through skin by imaging PW particles on skin

**Scheme 1:**
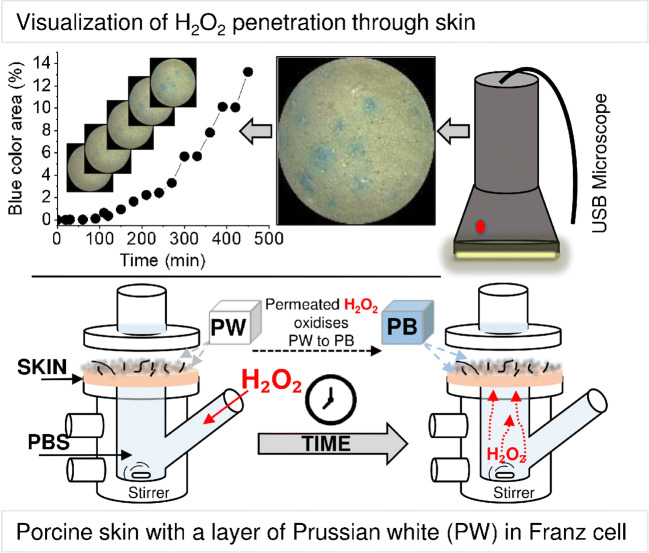
Schematic presentation of a skin membrane enclosed in a Franz cell. The skin was covered by PW microparticles. The lower chamber was filled with PBS containing H_2_O_2_ and 14 mM NaN_3_. With time, the H_2_O_2_ permeated the skin membrane and converted PW to PB. The development of blue colour was photographed using a USB microscope. The microscopy images revealed dominant H_2_O_2_ permeability pathways and the development rate of the blue area could be estimated

PW particles were deposited on the surface of the membrane (Scheme 1). Dermatomed (thickness 0.5 mm) or full-thickness (thickness 3–4 mm) skins were mounted in vertical-type Franz cells with the 6-mL lower chamber (serving as donor) filled with PBS, pH 7.4, 14 mM NaN_3_ (20 mM for full-thickness skin). The top chamber was filled with an identical solution (0.6 mL). The dermal skin side faced the lower chamber. Skins were left to equilibrate for 30 min in the Franz cell, and the electrical impedance was measured as described above. After equilibration, the PBS solution in the top chamber (stratum corneum plane of the skin) was removed and the skin surface structure was imaged using a digital USB microscope (× 1000, China). Upon emptying of the top chamber, the Franz cell position remained fixed throughout the experiment. Two milligrammes of PW suspended in 200 μL of 0.1 M KCl was spread on the skin surface and a defined amount of H_2_O_2_ was pipetted into the lower chamber of the Franz cell. The resulting solution (4 mM H_2_O_2_) in the lower chamber was continuously agitated using a magnetic stirrer. Directly after the H_2_O_2_ introduction, the skin surface with deposited PW particles was imaged and the photo was assigned as the skin/PW image at time 0 (0 min). The images were then recorded at regular time points. To overcome the disturbance from external light, an illumination ring (Optica Microscopes, Ponteranica, Italy) was mounted on the microscope and the entire Franz cell–microscope set-up was shielded from external light by a cardboard box.

Penetration experiments with a lower H_2_O_2_ concentration (0.5 mM) were supplemented by an external peristaltic pump (LabKemi, Sweden) connected to the lower chamber of the Franz cell. This allowed refreshing the PBS buffer/0.5 mM H_2_O_2_ each hour, to avoid possible degradation of H_2_O_2_. All measurements were conducted at room temperature, 22 °C.

### Image analysis

The ImageJ software (v. 1.51j8, Java 1.8.0_112, 64-bit) in combination with an adapted script (provided by ImaGene-iT, Lund, Sweden) was used for image processing. Briefly, skin surface images were cropped and converted to cyan, magenta, yellow, and black (CMYK) images. Then, cyan images were compared with an original photographic image; the B&W threshold was adjusted for each image (the threshold for different images varied slightly).

## Results

### Franz cell-based set-up for assessing epidermal sensing of H_2_O_2_

Taking into account that Prussian blue (PB) is highly biocompatible and nontoxic (an oral intake of 1–10 g/day of PB is recommended after exposure to radioactive caesium) [31, 32], we considered the possibility to monitor H_2_O_2_ on the skin surface using the Prussian white–Prussian blue redox couple [33]. Equation 1 describes the PW/PB redox conversion in reaction with H_2_O_2_.

1 $$ \mathrm{PW}\ \left({\mathrm{K}}_4{{\mathrm{Fe}}^{\mathrm{II}}}_4{\left[{\mathrm{Fe}}^{\mathrm{II}}{\left(\mathrm{CN}\right)}_6\right]}_3\right)+2\ {\mathrm{H}}_2{\mathrm{O}}_2\rightleftarrows \mathrm{PB}\ \left({{\mathrm{Fe}}^{\mathrm{II}\mathrm{I}}}_4{\left[{\mathrm{Fe}}^{\mathrm{II}}{\left(\mathrm{CN}\right)}_6\right]}_3\right)+4{\mathrm{O}\mathrm{H}}^{-}+4{\mathrm{K}}^{+} $$

H_2_O_2_, in the redox reaction (Eq. 1), is an oxidant that converts PW (K_4_Fe^II^_4_[Fe^II^(CN)_6_]_3_) to PB (Fe^III^_4_[Fe^II^(CN)_6_]_3_), and consequently the PW crystal colour changes from white to blue [34]. Hence, PW microcrystals were deposited on skin membrane surfaces in an attempt to verify if the conversion to PB can be used to monitor H_2_O_2_ penetration through skin membranes. This in vitro approach can be considered as a good model for assessing epidermal sensing of H_2_O_2_; we consider the approach as being a necessary step before testing the sensing in vivo using living animals or humans.

The experimental set-up comprised a skin membrane placed in a Franz cell (Scheme 1). The skin membrane was covered with a layer of PW crystals (3.1 mg cm^−2^). H_2_O_2_ permeability through skin resulted in PW turning blue. The colour change was visualised by a USB microscope. The results revealed that PW microparticles could be adopted as a sensing material for monitoring H_2_O_2_ penetration through skin. The observed H_2_O_2_ permeability was rationalised by considering transdermal penetration pathway theory [35].

### Inhibition of catalase activity in skin

In healthy skin, catalase, the main H_2_O_2_ detoxifying enzyme, converts H_2_O_2_ into water and oxygen [36]. In in vivo situations, when the skin is affected by various skin disorders (vitiligo [19], xeroderma pigmentosum [22] etc.), catalase activity is downregulated resulting in elevated H_2_O_2_ concentrations in the epidermis. Working with skin in vitro and aiming to monitor H_2_O_2_ presence on the skin surface, or to monitor H_2_O_2_ transdermal penetration, the skin catalase is an obvious problem since it converts H_2_O_2_ to H_2_O and O_2_. Therefore, to assure H_2_O_2_ penetration, skin catalase in skin membranes has been inhibited. The inhibition process was confirmed by using skin membranes firmly attached to oxygen electrodes. The set-up is known as skin-covered oxygen electrode (SCOE), which measures O_2_ liberated by skin catalase when the electrode is exposed to a solution of H_2_O_2_ [25, 28, 37]. Figure S1 in the supporting information provides an example of the current response when the SCOE is exposed to H_2_O_2_ in the absence and in the presence of a catalase inhibitor, specifically sodium azide, NaN_3_. Amperometric measurements with the SCOE did show that 14 mM of NaN_3_ is sufficient to inhibit more than 90% of skin catalase. Sufficient NaN_3_ amounts were thus always present in solutions of the Franz cell experiments.

### Synthesis and choice of Prussian white particles

Due to the potent diffusional barrier of the SC, permeability assays using Franz cells are usually conducted over several hours and it is expected that epidermal H_2_O_2_ monitoring in clinical situations might be equally time demanding. Hence, it is an absolute requirement that PW particles are stable against oxidation by ambient O_2_ and O_2_ dissolved in solution. PW oxidation by O_2_ is rarely reported, but depending on the particle structure, O_2_ can oxidise PW to PB in a few minutes or months. Various synthesis trials showed (data not shown) that micrometre-sized PW particles are more O_2_-stable. Specifically, we found that PW particles with an average size of 4.4 μm ± 1.2 μm (mean ± SD) (Fig. 1a) are O_2_-stable; when put in a closed Ependorf tube, or re-suspended in KCl solution, they can be kept in the PW state for weeks. Meanwhile, sub-micrometre particles, i.e. with an average size of 0.092 μm ± 0.027 μm (mean ± SD) (Fig. 1b), were unacceptably sensitive to O_2_, i.e. PW nanoparticles were converted to PB in a few minutes or hours.

These visual observations of PW particle stability towards O_2_ can be supported by the results of Hu and co-workers (2011), who performed Mössbauer spectroscopy measurements on the PW particles synthesised by the same synthesis protocol that was used in our study. In their work, Mössbauer spectra of PW particles were unchanged after exposure to air for 2 months [30]. The spectra show that there are just two absorption peaks, characteristic of the reduced form of iron, Fe^II^, indicating that PW of this size is stable against oxidation by O_2_ in air.

The size stability of our PW particles over time was assessed using light microscopy. A few PW particles were re-suspended in KCl solution and were imaged every day for 5 days. With no apparent change in shape and size of the particles, it was concluded that the PW particles are stable for at least 5 days in KCl solution (Fig. S4 and Table S2). In addition, SEM images of PW particles taken a year after synthesis did not show any obvious differences regarding size and/or shape, compared to the size and shape of the particles assessed by SEM directly (a few days) after synthesis (data not shown).

### PW/PB-based epidermal monitoring of H_2_O_2_ penetration through skin membranes

**Fig. 2:**
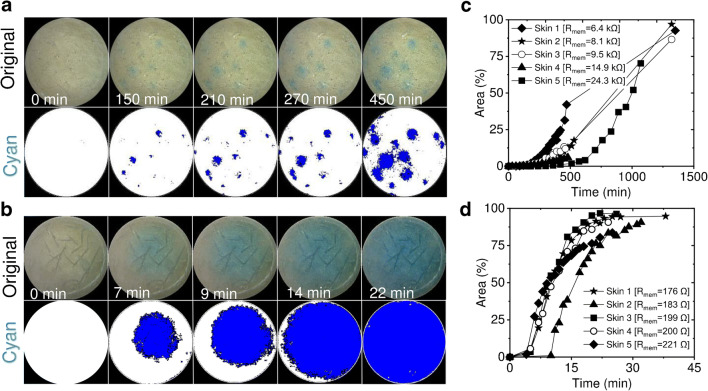
Visualisation of H_2_O_2_ skin membrane penetration by imaging the development of blue colour in the PW particle layer situated on the SC side of the skin membrane. The original photographic and corresponding cyan images are presented at certain time points after initiation of H_2_O_2_ penetration. Time 0 corresponds to the moment when the concentration of H_2_O_2_ in the lower Franz cell chamber was raised to 4 mM. Images taken for **a** unprocessed/natural skin membrane and **b** skin membrane after lipid extraction. The blue colour development is due to skin penetration of H_2_O_2_ and conversion of PW to PB (reaction Eq. 1). The images are taken by USB microscope. **c**, **d** Area % vs. time plots reflect an increase of the blue colour fraction with time due to PW conversion to PB on skin for cases of **c** unprocessed and **d** lipid-extracted skin, respectively. 100% area corresponds to the geometric area of the skin membrane since the entire surface became blue at the end of the experiment

Colorimetric monitoring of transdermal H_2_O_2_ penetration was done by performing Franz cell permeation assays; vide supra, Scheme 1. The eventual settling composed of the PW microparticle layer on a skin membrane with a 3.1 mg cm^−2^ PW coverage. The skin membrane was periodically photographed, which allowed monitoring of PW oxidation to PB due to the transdermal permeation of H_2_O_2_ from the dermal side of skin membrane onto its SC surface (Fig. 2).

The analysis of the images (Fig. 2) was carried out by converting the photographic skin surface images into cyan images and, subsequently estimating the percentage of the cyan colour area at the particular time point of the permeability experiments. As can be seen from Fig. 2a and c, the blue area increases over time, of H_2_O_2_ penetration experiment, albeit differently for five different skin membranes (the reasons are explained in the next paragraph). Control experiments with no addition of H_2_O_2_ into the Franz cells showed no PW oxidation to PB over 24 h.

As can be judged from longer measurements (> 24 h, Fig. 2c), most of the skin membranes approached 100% blue coverage indicating that PW particles were sufficiently evenly distributed over the entire surface of the skin membranes. This is particularly clear from the blue colour development of lipid-extracted skin membranes (Fig. 2b and d).

**Fig. 3:**
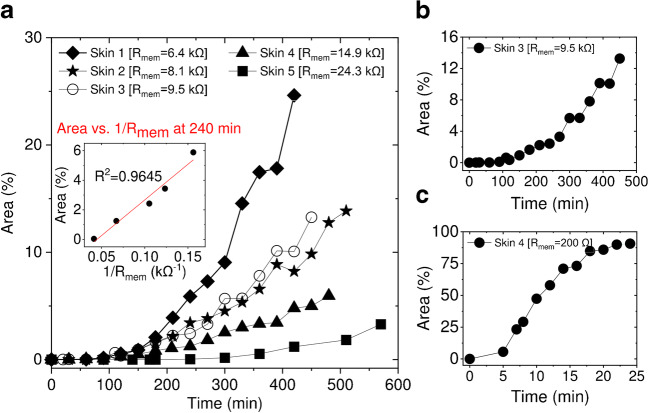
Development of the blue colour, area (%), on the skin surface due to H_2_O_2_ skin permeation. **a** The plot and the insert show that the development of the blue area strongly correlates with the reciprocal resistance of skin membranes (R_mem_). **b**, **c** Rate differences (compare time axes) of blue colour development on **b** untreated/natural and **c** lipid-extracted skin membranes

Prior to H_2_O_2_ penetration experiments, all skin membrane samples were evaluated by measuring the membrane electrical impedance, in order to estimate membrane resistance (R_mem_) values; an example of impedance data is shown in Fig. S2. Impedance measurements show that the resistance of untreated skin membranes, with a surface area of 0.64 cm^2^, was 12.6 kΩ ± 7.3 kΩ (mean ± SD, *n* = 5) on average, while lipid-extracted skins had an average resistance value of 0.20 kΩ ± 0.02 kΩ (mean ± SD, *n* = 5). As can be seen from Fig. 3a, the rate of blue colour development differs from membrane to membrane. The development of the blue area on skin correlated to the reciprocal resistance of the membrane (Fig. 3a, inset). For example, the blue colour fraction of the area, measured at the 4th hour (Fig. 3a, inset), was 5.9% for the membrane with R_mem_ = 6.4 kΩ, 3.4% for R_mem_ = 8.1 kΩ, 2.4% for R_mem_ = 9.5 kΩ, 1.2% for R_mem_ = 14.9 kΩ, and 0.04% for R_mem_ = 24.3 kΩ, respectively. The observed correlation between H_2_O_2_ permeation (i.e. blue colour development) across the skin membrane and reciprocal of skin resistance indicates that H_2_O_2_ penetrates the skin through the same pathway as hydrated ions. Additionally, the blue colour development kinetics on non-treated skin membranes, e.g. median resistance membranes (Fig. 3b, R_mem_ = 9.5 kΩ), was attenuated compared to lipid extracted membranes (Fig. 3c, R_mem_ = 0.2 kΩ). On lipid-extracted skin, more than 90% of the image did turn blue in less than 30 min (Fig. 3c). This confirms that SC lipid structures are the main barrier for permeability of hydrophilic biomarkers.

### Assessment of the mechanism of transdermal H_2_O_2_ penetration by epidermal sensing

**Fig. 4:**
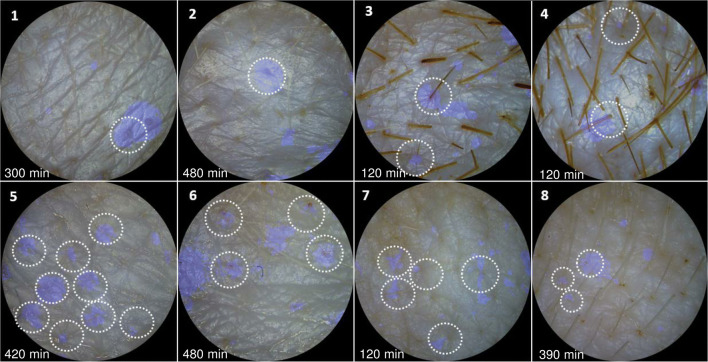
Overlay of skin surface images and blue (cyan) dot patterns developed in the PW layer on skin membranes during H_2_O_2_ permeability assays. Dashed circles mark identified hair shafts surrounded by oxidised PW, i.e. PB. The number on each sub-figure (1–8) indicates different skin membranes used for H_2_O_2_ permeability experiments. 1–3, 5, and 6 skin images are taken from H_2_O_2_ permeability experiments performed on split thickness (0.5-mm thickness) skin membranes when the concentration of H_2_O_2_ in the lower Franz cell chamber was raised to 4 mM. Skin image 4 and 7 is taken from the permeability experiment performed on split-thickness skin exposed to 0.5 mM H_2_O_2_. Skin image 8 is from a permeability experiment performed on a full-thickness skin membrane, exposed to 4 mM H_2_O_2_. The skin surface images were captured at the time points indicated on the image

During the PW-based epidermal monitoring of H_2_O_2_, a development of dot-like pattern of blue colour was always observed. This was conspicuously clear at the beginning (time < 4 h) of the experiments (Fig. 2a). This indicated that H_2_O_2_ permeates through skin appendages, and probably preferably though hair follicles. To check this hypothesis, all visible hair shafts were counted prior to the deposition of PW particles on skin. Then, skin images with marked hairs were overlaid with the blue (cyan) images (Fig. 4) and the percentage of blue colour associated with hair shafts was calculated (Table S3). Keeping the difficulty to recognise all hairs from photographic images in mind, the results are surprisingly consistent; 74% ± 7% (mean ± SD, *n* = 5) of the blue dots are associated with hair shafts (Table S3).

After membrane lipid extraction, the dot features of the blue colour were not observed (Fig. 2b); rather, the entire membrane area became blue, suggesting a complete disruption of H_2_O_2_ permeation pathways. These results indicate that hair follicles do not share the overall SC barrier features, and that is probably the reason why hair follicles act as dominant pathways of transdermal penetration of hydrophilic biomarkers such as H_2_O_2_. However, based on our calculations (Table S3), only 55% ± 22% (mean ± SD, *n* = 5) of all hair shafts were associated with blue colour development. The notable variation and relatively low fraction of hair follicles associated with facile H_2_O_2_ penetration (55%) might be due to structure and growth cycle differences of a particular hair. It is known that approximately 26% of the hair follicles present in human forearm skin are inactive; i.e. they do not excrete sebum and are filled with a corneocyte plug which restricts penetration of both hydrophilic and hydrophobic molecules [38]. The study however refers to human skin, but it is assumed that similar features are shared by porcine skin.

### Validation of the major H_2_O_2_ penetration pathway using full-thickness skin membranes

**Fig. 5:**
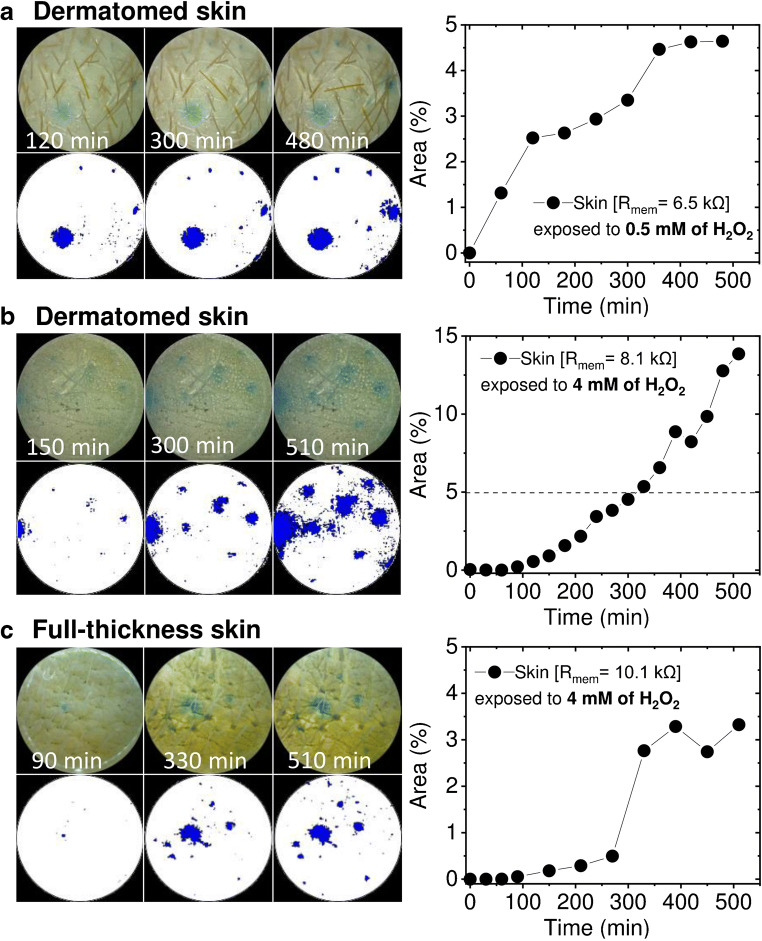
Epidermal monitoring of H_2_O_2_ penetration through skin membranes (Scheme 1). Skin membrane (0.5-mm thick) exposed to **a** 0.5 mM and **b** 4 mM H_2_O_2_. **c** Blue colour development on full-thickness skin (approx. 4-mm thick) during permeability of H_2_O_2_ from 4 mM solution

To validate that hair follicles are the dominant transdermal H_2_O_2_ penetration pathways, additional experiments were carried out. First, epidermal monitoring of the permeation from a lower, more physiologically relevant concentration (0.5 mM) of H_2_O_2_ was evaluated (Fig. 5a). The results (Fig. 5a; R_mem_ = 6.5 kΩ) indicate slower PW particle oxidation, compared to measurements on skin with similar resistance but with eight times higher H_2_O_2_ concentration (compared to Fig. 3a, skin1 with similar R_mem_ = 6.4 kΩ). When the skin membrane was exposed to 0.5 mM H_2_O_2_, after 8 h only 4.6% of the area was coloured blue (Fig. 5a), whereas in skin with similar resistance exposed to 4 mM of H_2_O_2_, 24.6% of the area was coloured after a similar time, i.e. 7 h (Fig. 3a). Again, the blue dot pattern is obvious even with the lower concentration of H_2_O_2_.

Additionally, since hair follicles can be lodged up to 4 mm deep inside the skin [39] and our dermatomed (split thickness) membranes are 500 μm thick, it can be suspected that the dot pattern observed may be due to cutting of deeper grown hair follicles. To ensure that the blue dot pattern is not an artefact, full-thickness skin membranes were prepared and experiments identical to those of dermatomed skin were performed (Fig. 5c). As shown in Fig. 5, regardless of whether dermatomed or full-thickness skin was employed, the same dot-like PW oxidation to PB pattern due to H_2_O_2_ transdermal permeation was observed. It can also be noticed that the first few visible blue dots appear 1–2 h after the start of the H_2_O_2_ permeability assay (Figs. 2 and 5).

## Discussion

### Obstacles of PW/PB-based epidermal monitoring of H_2_O_2_ on skin

To establish image analysis–based epidermal monitoring of H_2_O_2_ permeation across skin membranes, a PW particle suspension was applied directly on the skin membrane secured in a Franz cell (Scheme 1). The idea draws on the insight that PW microparticles in contact with H_2_O_2_ turn blue, i.e. PW becomes blue PB (Eq. 1). Given that various skin ailments and disorders are characterised by H_2_O_2_ production, a monitoring approach based on an easily detected colour change seemed obvious. However, two critical obstacles had to be circumvented. Firstly, skin is abundant with catalase; thus, to observe H_2_O_2_ penetration through skin, skin catalase needs to be inhibited. Secondly, it appeared that O_2_, present in air or as a solute, oxidises PW to PB. Though PW/PB-based H_2_O_2_ sensing is broadly used, the reactivity of PW with O_2_ is rarely reported [40–43].

High abundance of catalase in skin has been clearly shown by skin-covered oxygen electrode measurements [28]. Catalase in skin remains active after prolonged (more than a year) storage of skin membranes at − 20 °C (unpublished data); hence, for performing H_2_O_2_ permeability experiments, skin catalase must be inhibited. We found that 14 mM NaN_3_ is sufficient to inhibit catalase in skin (Fig. S1). Thus, appropriate amounts of NaN_3_ were always present in solutions used for H_2_O_2_ permeation measurements.

Another observation made during our studies was that the PW/PB redox pair has a considerable sensitivity to O_2_. This fact is often neglected since sensors for H_2_O_2_ based on PW/PB are usually tested for several minutes only. However, transdermal permeation might take hours; the long time is needed for molecules (especially hydrophilic) to cross the barrier maintained by the SC. Despite the fact that many research articles [44, 45] and detailed reviews [34, 45] focus on PB synthesis, only a few reports can be found that address synthesis of PW with reduced reactivity towards O_2_ [30, 46]. The method proposed by Hu and Jiang [30] yielded PW cube-like microparticles (Fig. 1a), and satisfactory stability against oxidation by O_2_; the PW microparticles stay white (i.e. white-grey) for more than 2 months when kept in a closed Eppendorf tube. Based on experience with different synthetic routes, it was concluded that smaller particles (< 0.2 μm) are highly prone to oxidation by O_2_ in air or solution, and are thus not suitable for optical epidermal sensing of H_2_O_2_. Thus, micrometre-sized PW particles were used to develop epidermal H_2_O_2_ sensing and to visualise pathways of H_2_O_2_ penetration through skin.

### Epidermal monitoring of biomarkers can benefit from skin resistance measurements

Epidermal monitoring of transdermal H_2_O_2_ penetration carried out in this work relied on (i) skin membranes mounted in Franz cells; (ii) deposition of a H_2_O_2_-sensitive, but O_2_-insensitive PW microparticle layer on the SC face of skin membranes; (iii) recording the blue colour development of the dispersed PW microparticles, due to reaction with permeated H_2_O_2_; and (iv) complementing the colour-based assay with skin integrity measurements using electrical impedance spectroscopy. Repeated epidermal monitoring of H_2_O_2_ with different skin samples revealed that it takes 3 to 10 h until about 4% of the skin area is covered with blue colour (Fig. 3a). The extended time required for H_2_O_2_ penetration confirms that the SC is a potent diffusional barrier for the hydrophilic biomarkers. The results further suggest that clinically relevant epidermal sensing of H_2_O_2_ might require robust hour-long skin monitoring. It is important to notice that reciprocal of skin resistance, 1/R_mem_, i.e. conductance, correlated linearly with the blue area development rate (Fig. 3a, inset). The simultaneous R_mem_ and H_2_O_2_ permeability measurements add to the understanding of the differences observed in H_2_O_2_ penetration through different skins and help explain the biological variability of the SC biobarrier. Summarising, it can be concluded that if complemented with simultaneous skin impedance measurements, epidermal sensing of biomarkers, as in this case H_2_O_2_, should significantly improve analysis robustness and result interpretation. We believe that such a combination has not yet been realised in epidermal sensing.

### Epidermal monitoring of H_2_O_2_ revealed hair follicles as the dominant H_2_O_2_ permeability pathways through skin

In a simplified view of permeation across a skin barrier, at least two separate routes of penetration can be distinguished. First, skin appendages comprised sweat ducts and hair follicles, and, second, intercellular and transcellular penetration pathways [47–49]. The scientific community questions if skin appendages have an impact on transdermal permeation, owing to the minute skin surface fraction occupied by the appendages, i.e. approx. 0.1% of the total skin surface [49]. In addition, the molecular details of how hair follicles and appendages allow higher permeability remain unclear. Some researchers state that due to the sebum present in active hair follicles, this route facilitates lipophilic molecule penetration [50, 51]. Another group of scientists suggests that hair follicles are the dominant transdermal penetration pathways for hydrophilic molecules [52–54]. The last rationale was recently pointed out in epidermal measurements of interstitial glucose [17]. The researchers compared glucose detection on a skin area with high hair follicle density (34 hairs/cm^2^) vs. an area with low density of hair follicles (6 hairs/cm^2^). They found that the follicle-dense skin allowed almost nine times higher flux of glucose if compared with the flux across the follicle-poor area of the porcine skin [17].

Our results show that H_2_O_2_ penetration through skin generates a blue colour dot-like pattern in the H_2_O_2_-sensitive PW particle layer deposited on the surface of skin membranes (Figs. 2, 4, and 5). The pattern strongly supports the appendage route as being the dominant transdermal H_2_O_2_ penetration pathway. Overlaying of skin images, recorded before deposition of PW layer (Fig. 4), with the blue colour dot images of the same skin covered with PW during H_2_O_2_ penetration experiments, shows that a majority of the blue dots develop around the hair shafts (74% ± 7%, Table S3). This strongly suggests that hair follicles are the dominant H_2_O_2_ penetration pathways. A substantial number of blue colour dots (≤ 26%) appeared at skin sections without visible hair, which might be due to invisible smaller hairs or other appendages (e.g. sweat ducts). Even given this uncertainly, our results show that H_2_O_2_ penetration through skin is dominated by the hair follicle pathway. This strongly favours the argument that permeation of hydrophilic molecules via the hair follicle pathway is substantial and should be targeted by epidermal sensing [17, 52–54].

To reinforce the statement that H_2_O_2_ preferentially penetrates skin through hair follicles, the permeability experiments were performed using full-thickness skin (4 mm thick). The same blue dot pattern in the H_2_O_2_-sensitive PW microparticle layer developed (Fig. 5c). This confirms that the observed H_2_O_2_ penetration pattern is not due to dermatome-induced holes during preparation of thinner (0.5 mm) skin membranes.

Additionally, micrometre-sized particles (< 10 μm) can enter the follicular orifices [55]; hence, the PW particles, with an average size of 4.4 μm ± 1.2 μm (mean ± SD), may be localised inside the hair orifice/follicles, contributing to the development of the dot-like blue colour patterns observed during H_2_O_2_ penetration assays. In such a case, H_2_O_2_ penetrating the skin initially will meet PW particles lodged inside the hair follicle, explaining the observed blue dot pattern. As can be seen in Fig. 2b, after lipid extraction, no blue dot pattern was observed and H_2_O_2_ seems to cross the skin evenly/homogenously. Additional experiments have also been done by first depositing a filter paper on the skin membrane and then pipetting a suspension of PW particles on the top of the filter. Similar dot-like patterns (data not shown) of PB appeared even for this H_2_O_2_-sensing layer configuration. Altogether, the results strongly suggest that H_2_O_2_ has a strong preference to penetrate through the appendages of skin, particularly through hair follicles. This knowledge is important in the development of clinically robust epidermal sensing of H_2_O_2_ with micrometre resolution. Our results show that micrometre resolution would allow faster biomarker detection: probably in 1–2 h as judged from the appearance of the first blue dots in Figs. 2 and 5.

## Conclusions

This study aimed to visualise H_2_O_2_ penetration through skin membranes in order to model and rationalise strategies for the development of clinically relevant, epidermal sensing of ROS biomarkers, specifically H_2_O_2_. We conducted epidermal monitoring of H_2_O_2_ penetration through skin by using H_2_O_2_-sensitive Prussian white microparticles. The PW particle layer was deposited on the surface of skin membranes and after some time the PW particles turned blue, signalling H_2_O_2_ permeation through the skin. We found that PW particles with an average size of 4.4 μm ± 1.2 μm (mean ± SD) were sufficiently O_2_-insensitive to allow prolonged (at least up to 24 h) epidermal monitoring of H_2_O_2_. The monitoring of H_2_O_2_ penetration was only possible after inhibition of skin catalase. Unfortunately, in most clinical cases, skin catalase inhibition will not be allowed; however, diseased skin is usually characterised by downregulation of catalase. Thus, epidermal sensing of H_2_O_2_ in clinically relevant situations might still be possible with a PW microparticle layer on the skin. Additionally, we found that the penetration of H_2_O_2_ through skin generates a blue dot pattern in the PW layer. About 74% ± 7% of the dots were associated with visible hair shafts. This observation, for the first time, firmly establishes that hair follicles are the dominant H_2_O_2_ penetration pathways in skin. In general, the results suggest that targeting hair follicles with micrometre resolution should provide more sensitive and rapid monitoring of low molecular weight, hydrophilic biomarkers of skin disorders. This is especially important to realise for development of epidermal sensing of H_2_O_2_. H_2_O_2_ is one of the common ROS, elevated in many inflammatory and autoimmune skin disorders, and its epidermal monitoring would be extremely valuable in managing these pathological conditions.

## Supplementary Information

ESM 1 (PDF 1238 kb)

ESM 2 (MP4 97,414 kb)
