# Supplementary material for: Visualisation of H2O2 penetration through skin indicates importance to develop pathway-specific epidermal sensing
Source: Mikrochim Acta. 2020 Nov 13;187(12):656. doi: 10.1007/s00604-020-04633-9 (PMC7666278; doi:10.1007/s00604-020-04633-9)
Supplement: Supplementary file 1 — (PDF 1238 kb) [file 604_2020_4633_MOESM1_ESM.pdf]

Electronic Supplementary Material

Visualization of H_2O_2 penetration through skin indicates importance to develop pathway-specific epidermal sensing

Skaidre Jankovskaja[1,2], Anaïs Labrousse[3], Léa Prévaud[4], Bo Holmqvist[5], Anders Brinte[5], Johan Engblom[1,2], Melinda Rezeli[6], György Marko-Varga[6], and Tautgirdas Ruzgas[1,2]

[1]Department of Biomedical Science, Faculty of Health and Society, Malmö University, 205 06 Malmö, Sweden
[2]Biofilms - Research Center for Biointerfaces, Malmö University, 205 06 Malmö, Sweden
[3]Department of Biological Engineering, Clermont Auvergne University, 63100 Aubiere, France
[4]Faculty of Sciences, University of Montpellier, 34085 Montpellier, France
[5]ImaGene-iT, Medicon Village, 223 81 Lund, Sweden
[6]Clinical Protein Science & Imaging, Biomedical Centre, Department of Biomedical Engineering, Lund University, BMC D13, 221 84 Lund, Sweden
Corresponding author tautgirdas.ruzgas@mau.se

Table of contents

Fig. S1: Evaluation of catalase activity in skin by amperometric measurement with skin membrane covered oxygen electrode (SCOE) exposed to H_2O_2 and NaN_3.
Fig. S2: Evaluation of skin membrane integrity by measurements of electrical impedance spectroscopy.
Fig. S3: Assessment of Scanning electron microscopy image to estimate Prussian white (PW) particles shape and size distribution
Fig. S4: Light microscopy images of Prussian white (PW) particles over time.
Table S1: Prussian white (PW) particle characterization, evaluation of size and shape distribution
Table S2: Prussian white (PW) particles side length measured over time.
Table S3: Summary accounting hair follicle contribution to H_2O_2 penetration through skin.

The **video of skin membrane preparation** is submitted as electronic supporting information.

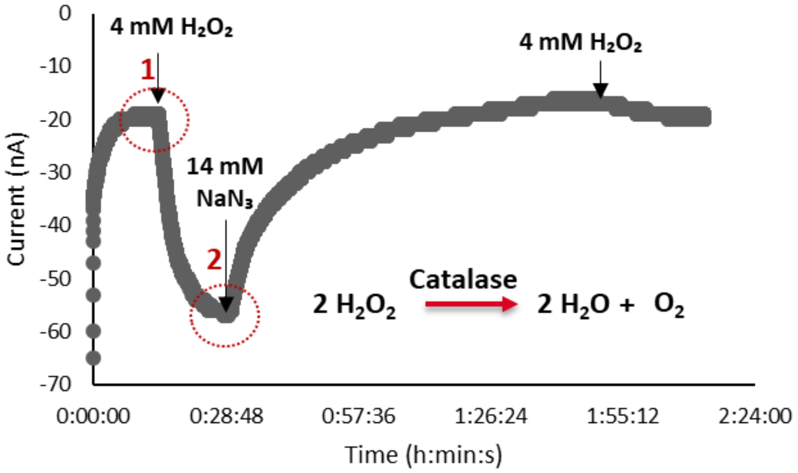

Fig. S1. Evaluation of catalase activity in skin by amperometric measurement with skin membrane covered oxygen electrode (SCOE) exposed to H_2O_2 and NaN_3. Current response of the SCOE vs time indicating when different compounds were added into the measurement cell. Time interval 0 to “1”: stabilization of SCOE current after immersed in PBS. At time point indicated as “1” (00:14:27) the current response (caused by O_2 generation in skin membrane) is due to addition of 4 mM of H_2O_2, i.e., H_2O_2 penetrates into the skin where it is quickly converted to H_2O and O_2 by catalase naturally present in skin. The response decreases (“2”, 00:30:00) after addition of catalase inhibitor NaN_3 (14 mM).

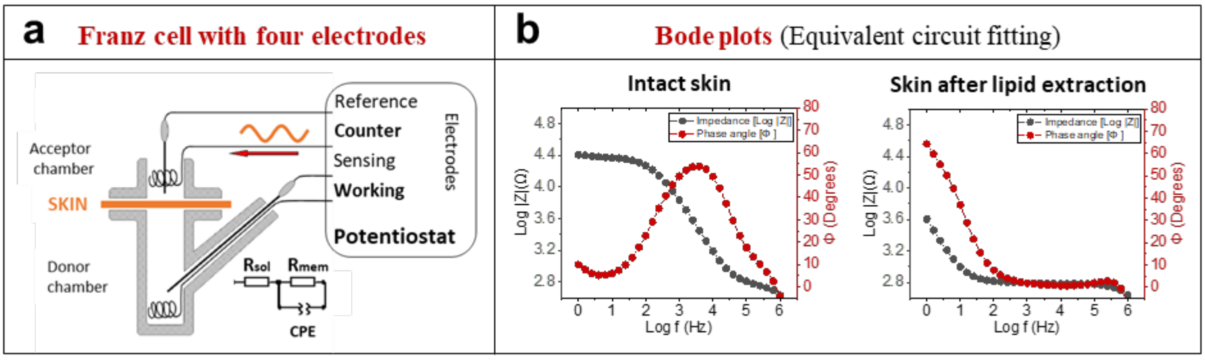

Fig. S2. Evaluation of skin membrane integrity by measurements of electrical impedance spectroscopy. (a) Franz cell equipped with four electrodes for measurements of electrical impedance spectroscopy including equivalent circuit used to fit the data and calculate skin membrane resistance, R_{mem}. (b) An example of impedance data (Bode plot) collected with intact skin membrane and skin membrane after lipid extraction. To derive R_{mem}, fitting of the data (b) to the equivalent circuit (a) was done by using software provided by Ivium Technologies BV.

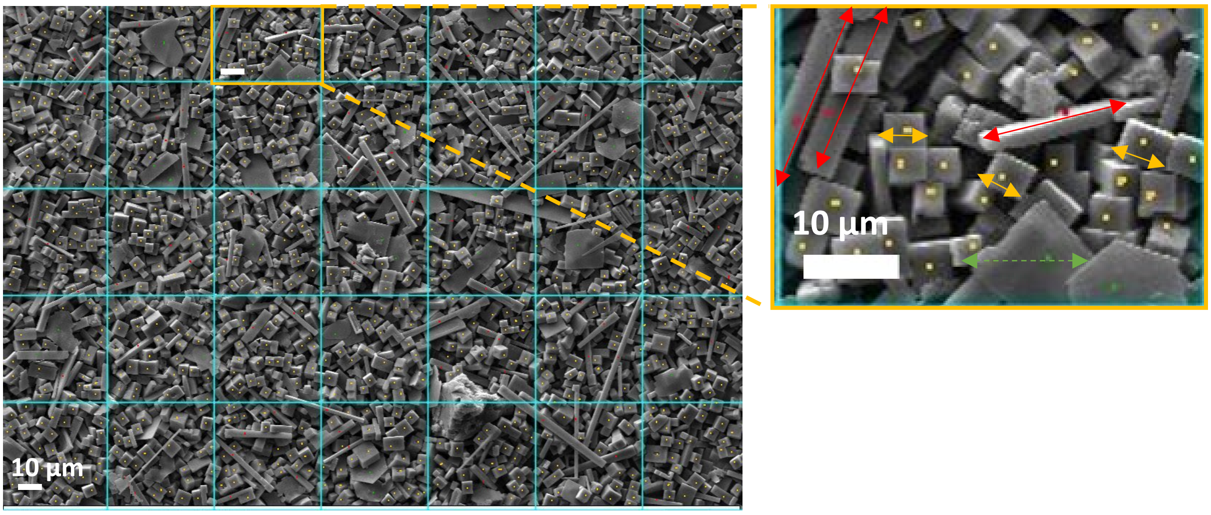

Fig. S3. Scanning electron microscopy image used to estimate Prussian white (PW) particle shape and size distribution (summarized in Table S1). PW particles were assigned to three different groups based on their shape: cubes, cuboids (length is at least 3 times bigger than width) and sheets. Cube or cuboid like PW particles were evaluated by measuring the longest side length (enlarged image: yellow arrows for cubes, red for cuboids). Sheets were assessed by measuring their diameter (green dashed arrow). In total, dimension of 1010 particles were measured with ImageJ program based on procedure as follows. Whole image was divided into 35 squares. PW particle size was measured in each square separately and then results were added and treated as one dataset.

Table S1. Prussian white (PW) particle characterization, size and shape distribution evaluation based on the image presented in Fig. S3 and Fig. 1.

**Particle shape**	**Dimensions, µm**			**Number**
	Mean±SD	Median	Mode	**(%)**
Cubes (n=910)	4.4±1.3	4.2	3.6	89
Cuboids (n=80)	20.7±11.6	17.6	14.3	8
Sheets (n=30)	13.6±9.1	11.7	5.9	3
Cubes + cuboids (n=990)	5.7±5.6 *(4.3±1.1)	4.3	4.6	9700%

[1]For cube and cuboid shape particles the biggest side length was measured and for sheets the diameter was measured.
[2]Value obtained after fitting particle size to LogNormal distribution model

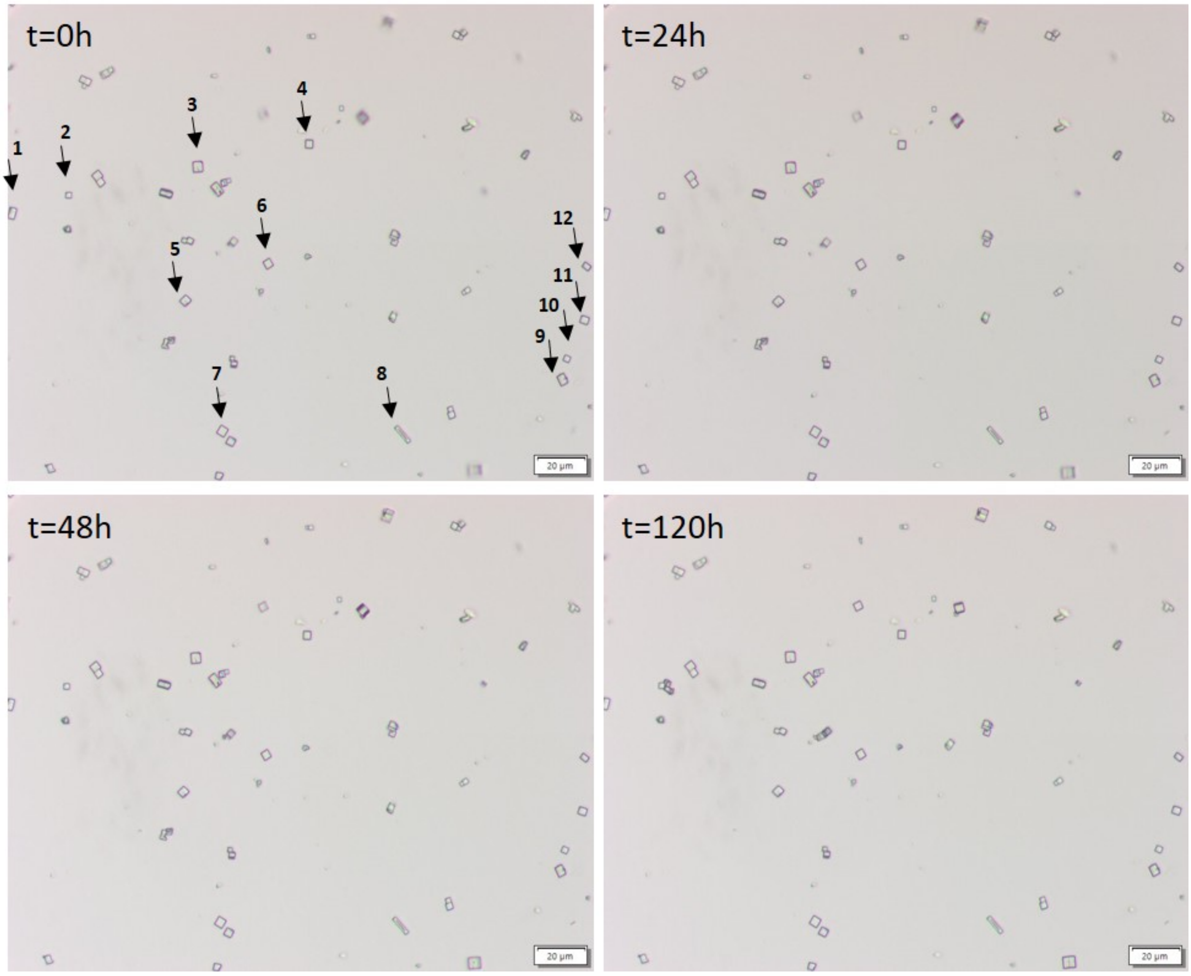

Fig. S4. Light microscopy images of Prussian white (PW) particles over time. PW particles (re-suspended in 0.1 M KCl solution) were deposited on the glass slide, covered with cover glass, sealed with nail polish and placed under the light microscope (fixed position) for five days. Time 0 h (t = 0 h) indicates the first image taken after PW sample preparation and image focusing (~30 min after PW particles re-suspension in KCl solution); subsequent images were taken at particular time points (24 h, 48 h and 120 h). No changes in PW particle shape or sizes (Table S2) were observed over 120 h time period.

Table S2. Prussian white (PW) particle side length measured over time of their incubation in PBS. Light microscopy images taken over time on the same PW sample area were evaluated by selecting 12 individual PW particles (Fig. S4; black arrows with numbers) and measuring their side length at different time points.

Particle No.	Particle side lenght (μm)		
	0 h	48 h	120 h
1	4.5	4.6	4.3
2	2.4	2.2	2.2
3	3.4	3.8	3.6
4	2.8	2.8	2.6
5	3.7	3.9	3.4
6	3	3.2	3.1
7	3.5	3.2	3.4
8	7.7	7.7	7.9
9	4.3	4.4	4.1
10	2.5	2.4	2.4
11	3.2	3	3.3
12	2.5	2.5	2.7

Table S3. Summary accounting hair follicle contribution to H_2O_2 penetration through skin.
Overlay of cyan (blue) dot pattern image and the skin surface image with counted hair shafts. Black dashed circle: blue colour associated with the hair shaft; yellow circle: blue dot without recognized hair. Images obtained during Franz cell setup based H_2O_2 permeability assay through porcine skin.

**Skin used (replicates)**	**Skin surface with counted hairs**	**Original skin surface image with deposited PW oxidised to PB by penetrated H_2O_2**	**Cyan image**	**Overlay image** (cyan+skin surface)	**Number of hairs counted**	**Blue colour developed around the recognized /counted hair**	**Blue 'dot' without associated hair**	**Blue dots (%) developed around hair shaft compared to total amount blue dots developed**	**Hair shafts (%) surrounded by blue colour vs total hairs counted on the skin**
**1**<br>7 h 40 min after H_2O_2 addition (0.5 mm thickness skin; 4 mM H_2O_2)	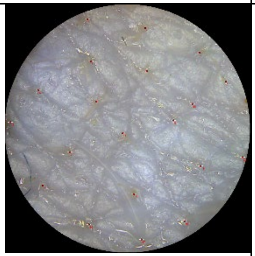	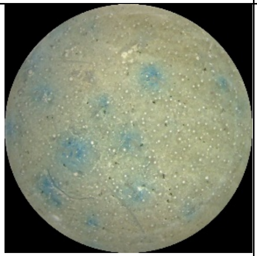	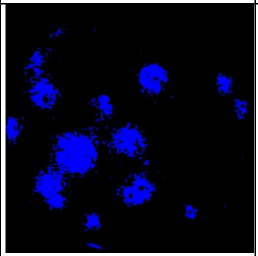	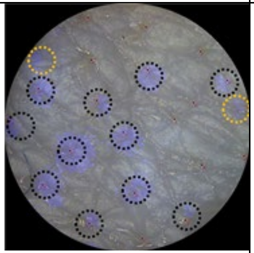	20	11	2	85%	55%
**2**<br>8 h 00 min after H_2O_2 addition (0.5 mm thickness skin; 4 mM H_2O_2)	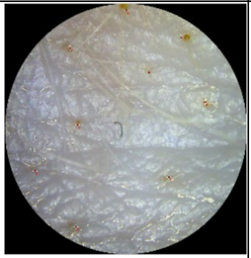	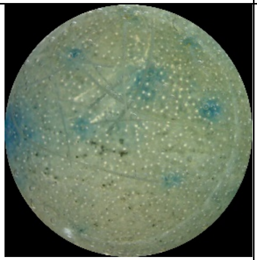	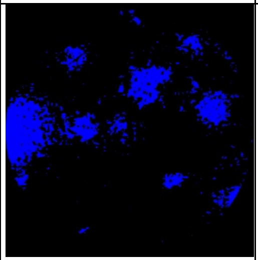	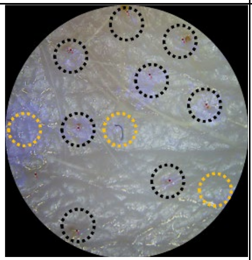	9	8	3	73%	89%
**3**<br>5 h 00 min after H_2O_2 addition (0.5 mm thickness skin; 4 mM H_2O_2)	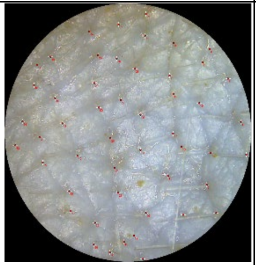	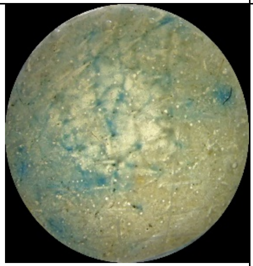	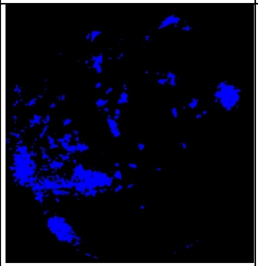	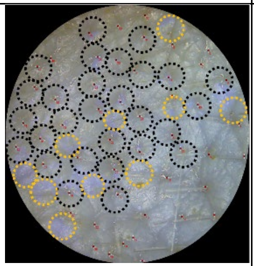	50	29	10	74%	58%

**4**<br>8 h 10 min after H_2O_2 addition (0.5 mm thickness skin; 4 mM H_2O_2)	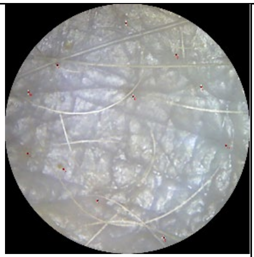	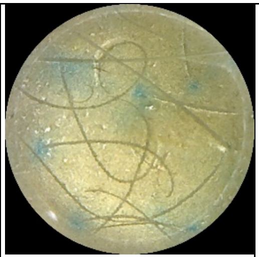	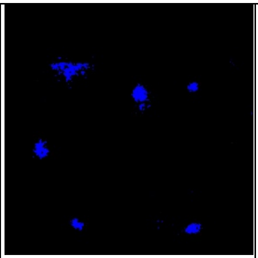	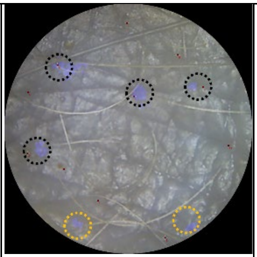	11	4	2	67%	36%
**5**<br>8 h 30 min after H_2O_2 addition (0.5 mm thickness skin; 4 mM H_2O_2)	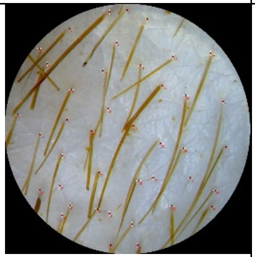	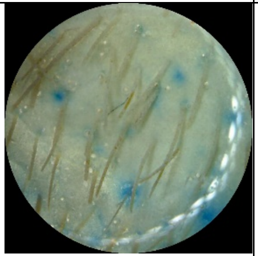	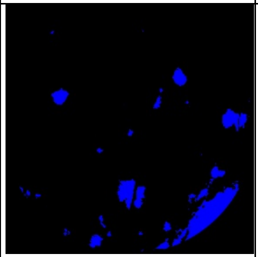	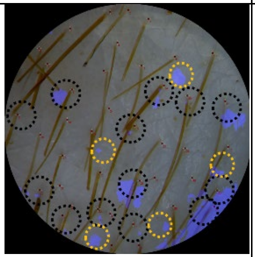	41	13	5	72%	37%
**Mean±SD (n=5)**								**74%±7%**	**55%±21%**
***6**<br>8 h 00 min after H_2O_2 addition (Full thickness skin; 4 mM H_2O_2)	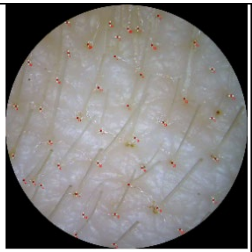	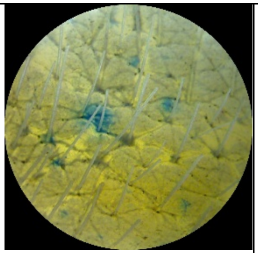	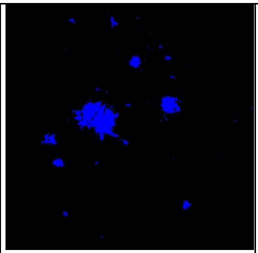	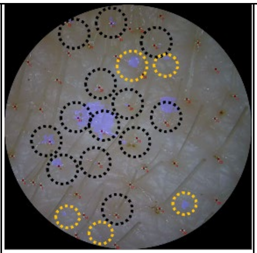	45	13	5	72%	29%
***7**<br>8 h 00 min after H_2O_2 addition (0.5 mm thickness skin; 0.5 mM H_2O_2)	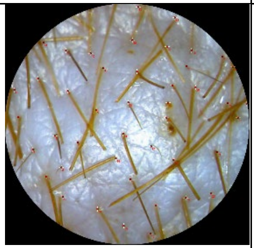	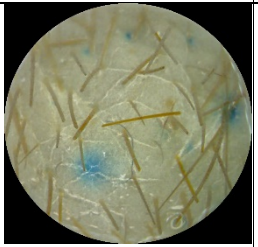	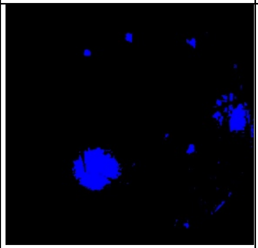	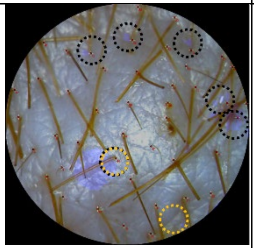	44	6	2	75%	14%

* Different conditions of the experiment compared to replicates **1-5**: 0.5 mm thickness skin exposed to 4 mM of H_2O_2. **6**: full thickness skin exposed to 4mM of H_2O_2; **7**: 0.5 mm thickness skin exposed to 0.5 mM of H_2O_2.
